# 治疗前血清载脂蛋白水平与小细胞肺癌患者预后的相关性研究

**DOI:** 10.3779/j.issn.1009-3419.2020.104.21

**Published:** 2020-10-20

**Authors:** 娅 董, 浩澄 王, 东凤 单, 林薇 张, 壮 于

**Affiliations:** 266003 青岛，青岛大学附属医院肿瘤科 Department of Oncology, The Affiliated Hospital of Qingdao University, Qingdao 266003, China

**Keywords:** 肺肿瘤, 载脂蛋白A-I, 载脂蛋白B/载脂蛋白A-I, 预后, Lung neoplasms, Apolipoprotein A-I, Ratio of apolipoprotein B to apolipoprotein A-I, Prognosis

## Abstract

**背景与目的:**

肺癌为癌症相关死亡的主要原因，在所有类型的肺癌中，小细胞肺癌（small cell lung cancer, SCLC）预后极差，本研究评估SCLC患者血清载脂蛋白水平与预后的关系，寻求指导SCLC诊治的新指标。

**方法:**

本研究回顾性分析了122例SCLC患者的临床资料。收集治疗前2周内血清载脂蛋白水平患者的临床结果，主要包括载脂蛋白（apolipoprotein, Apo）A-I、ApoB以及ApoB/ApoA-I。患者的无进展生存期（progression free survival, PFS）和总生存期（overall survival, OS）是主要的结局指标。用X-tile工具确定各指标的最佳临界值，生存分析采用*Kaplan-Meier*法分析，采用*Cox*回归分析法进行单因素分析和多因素分析。

**结果:**

与低ApoA-I水平组的患者相比，高ApoA-I水平组（ApoA-I > 1.12 g/L）的患者具有更好的OS（21.5个月*vs* 12.3个月，*P*=0.007）和PFS（7.3个月*vs* 5.5个月，*P*=0.017）。相反，具有较高ApoB/ApoA-I水平的患者比ApoB/ApoA-I水平较低的患者的中位OS差（13.4个月*vs* 20.7个月，*P*=0.012）。多因素*Cox*回归分析表明，ApoA-I是影响SCLC患者PFS的独立预后因素（HR=0.67, 95%CI: 0.45-0.99, *P*=0.043）。ApoB/ApoA-I是影响SCLC患者OS的独立危险因素（HR=1.98, 95%CI: 1.21-3.23, *P*=0.007）。

**结论:**

治疗前血清ApoA-I水平和ApoB/ApoA-I水平可能是SCLC的重要预后因素，有助于判断患者的预后。

## Abstract (en)

**Background and objective:**

Lung cancer is the leading cause of cancer-related death, and small cell lung cancer (SCLC) has a poor prognosis in all types of lung cancer. This study evaluated the relationship between pretreatment serum apolipoprotein levels and prognosis in patients with SCLC, seeks a new index can guide diagnosis and treatment of SCLC.

**Methods:**

This study retrospectively analyzed the clinical data of 122 patients with SCLC. The clinical results of patients with serum apolipoprotein levels within 2 weeks before treatment were collected, including apolipoprotein AI (ApoA-I), apolipoprotein B (ApoB), and the ratio of apolipoprotein B to apolipoprotein AI (ApoB/ApoA-I). Patients' progression-free survival (PFS) and overall survival (OS) are the main outcome indicators. The best critical to determine the index's value by X-tile tool. For survival analysis, *Kaplan-Meier* method was used for analysis, and *Cox* regression analysis method was used for single factor analysis and multifactor analysis.

**Results:**

Compared with patients with low ApoA-I levels, patients with high ApoA-I levels (ApoA-I > 1.12 g/L) had better OS (21.5 mon *vs* 12.3 mon, *P*=0.007) and PFS (7.3 mon *vs* 5.5 mon, *P*=0.017). In contrast, patients with higher ApoB/ApoA-I levels had worse median OS than patients with lower ApoB/ApoA-I levels (13.4 mon *vs* 20.7 mon, *P*=0.012). Multivariate *Cox* regression analysis showed that ApoA-I was an independent prognostic factor affecting PFS in SCLC patients (HR=0.67, 95%CI: 0.45-0.99, *P*=0.043). ApoB/ApoA-I is an independent risk factor for OS in patients with SCLC (HR=1.98, 95%CI: 1.21-3.23, *P*=0.007).

**Conclusion:**

Serum ApoA-I level and ApoB/ApoA-I level before treatment can be important prognostic factors for SCLC, which is helpful to judge the prognosis of patients.

肺癌是威胁人群健康和生命的最常见的恶性肿瘤之一，肺癌的发病率和死亡率逐年增加。根据组织病理学类型不同，肺癌可分为小细胞肺癌（small cell lung cancer, SCLC）和非小细胞肺癌（non-small cell lung cancer, NSCLC）。SCLC约占肺癌的14%-20%，其特点是加倍时间短、侵袭性强、转移趋势强^[1-3]^。尽管SCLC对化学疗法和放射疗法敏感，但患者一线治疗失败，出现复发或进展，再次治疗预后极差。因此，早期评估疗效和预期生存率可以极大地帮助和指导临床治疗。近年来，一些回顾性研究^[4, 5]^表明，某些血液学预处理参数可能会影响SCLC患者的预后，但是，明确新的独立预后因素也很重要，这将有助于我们更好地指导SCLC患者的治疗。

一些研究^[6, 7]^表明血脂水平与癌症之间存在相关性。一项大型队列研究^[8]^表明，总胆固醇、甘油三酸酯和低密度脂蛋白（low density lipoprotein, LDL）与男性肺癌有关。也有回顾性研究^[9]^表明，血清LDL水平是SCLC患者的独立危险因素。高密度脂蛋白（high density lipoprotein, HDL）与NSCLC预后之间的关系有相关报道^[10]^。然而，SCLC患者血清载脂蛋白水平与预后之间的关系尚未有研究。载脂蛋白（apolipoprotein, Apo）通过结合和转运甘油三酸酯、总胆固醇和磷脂在调节血脂平衡中起重要作用，还广泛地参与肿瘤的发生和发展^[11]^，载脂蛋白参与肿瘤发生和发展的方式可能是通过促进肿瘤的侵袭和转移、促进抗肿瘤药物的传递和直接的氧化应激反应^[12-16]^。在临床工作中，我们经常用来反映患者载脂蛋白水平的生物标志物主要是载脂蛋白ApoA-I、ApoB以及ApoB/ApoA-I。目前已发表的研究^[17-19]^表明，ApoA-I的水平与多种癌症（例如胃癌、鼻咽癌、乳腺癌和结直肠癌）的存活率有关。Shi等^[20]^主要收集SCLC患者的肿瘤组织，用来测定肿瘤细胞胞质中的ApoA-I浓度，他们发现，ApoA-I和ApoE的表达在复发患者和非复发患者中是不同的，结果表明，两种载脂蛋白可能对SCLC的预后具有预测作用，但是治疗前血清载脂蛋白的水平与SCLC预后之间的关系尚不清楚，本研究通过分析SCLC患者的临床资料，探讨了患者初诊时的血清载脂蛋白水平与其预后的关系。

## 1 材料与方法

### 1.1 患者筛查标准

我们回顾性收集了2015年1月-2018年12月在单一三级癌症中心接受治疗的SCLC患者的基本数据。纳入标准：①初次接受诊治的患者，并且通过组织病理学或细胞学诊断确诊为SCLC；②患者的治疗前实验室检查数据来自同一检测机构；③东部肿瘤协作组（Eastern Cooperative Oncology Group, ECOG）评分在0分-3分之间。排除标准：①既往有原发肿瘤部位的手术史或粒子植入史；②过去或当前同时患有其他恶性肿瘤；③服用降脂药物、治疗糖尿病药物或皮质类固醇；④随访数据不完整。

### 1.2 资料收集

我们从患者电子病历系统中获取了纳入患者的临床资料，包括患者的人口学特征、吸烟史、肿瘤学分期、治疗方案及以患者身高体重计算的身体质量指数（body mass index, BMI）。我们主要收集的血清学生物标志物结果包括ApoA-I、ApoB、ApoB/ApoA-I和脂蛋白a。根据美国退伍军人肺癌协会（Veterans Administration Lung Study Group, VALSG）分期系统确定SCLC分期。

### 1.3 疗效评估

入组患者每2个治疗周期后，使用动态计算机断层扫描（CT）仔细评估疗效。在完成抗肿瘤治疗后2年内，每3个月随访一次，3年-5年内每6个月一次，此后每年一次。每次随访均行胸部计算机断层扫描（computed tomography, CT）或正电子发射型计算机断层显像（positron emission computed tomography, PET）扫描。根据实体瘤反应评估标准（Response Evaluation Criteria In Solid Tumors, RECIST）1.1版进行肿瘤反应评估。

### 1.4 随访及观察指标

通过患者定期到院检查结果及电话咨询方式随访，最后随访时间为2019年12月31日。主要观察指标为患者的无进展生存期（progression free survival, PFS）和总生存期（overall survival, OS），PFS定义为接受治疗开始到观察到疾病进展或者发生因为任何原因的死亡之间的这段时间，OS定义为从病理诊断到任何原因死亡或最后一次随访的时间。

### 1.5 统计分析

使用X-tile工具测定ApoA-I、ApoB、ApoB/ApoA-I和脂蛋白a的最佳临界值。我们使用*Mann-Whitney U*检验或*Fisher*精确检验来检测不同ApoA-I组、ApoB/ApoA-I组间患者基本特征之间的差异，采用*Kaplan-Meier*法绘制不同分组患者的生存曲线，采用*Log-rank*检验比较曲线之间的差异。采用*Cox*回归模型确定风险比（hazard ratio, HR），将所有变量纳入在单因素*Cox*回归模型中，*P* > 0.1被认为是重要的变量，进而将重要的变量纳入多因素*Cox*回归模型中评估，以确定独立变量，而风险比以相应的95%置信区间被报道为相对可信，所有统计分析均采用SPSS 25.0，所有统计检验均为双侧检验，*P* < 0.05为差异有统计学意义。

## 2 结果

### 2.1 患者的基本特征

患者的基本特征在表 1中详述。该研究纳入了122例患者，他们的平均年龄为61岁，其中男性患者占大多数（共102例患者，占84%），并且大多数患者有吸烟史（89例），71例患者处于局限期，51名患者在诊断时已出现远处转移，处于广泛期。绝大多数患者（104例）接受了依托泊苷类化疗，少部分患者（18例）接受伊立替康方案化疗，其中有56例患者接受过胸部放疗，其中有46例患者处于局限期，10例患者处于广泛期。其中仅有5例患者化疗的同时同步放疗，剩余患者在放疗结束后序贯化疗。66例患者未曾接受过任何放疗。计算患者的BMI，其中BMI≥24 kg/m^2^的患者共有67例，BMI < 24 kg/m^2^的患者共有55例。本研究中位随访时间为19.3个月（范围12.3个月-35.8个月）。

**1:** 患者的基本特征和不同ApoA-I、ApoB/ApoA-I分组与各临床因素的关系 The basic characteristics of patients and the relationship between different ApoA-I, ApoB/ApoA-I groups and clinical factors

Characteristic	All patients (*n*=122)	ApoA-I > 1.12 g/L (*n*=82)	ApoA-I≤1.12 g/L (*n*=40)	*P*	ApoB/ApoA-I > 0.97 (*n*=33)	ApoB/ApoA-I ≤0.97 (*n*=89)	*P*
Demographics							
Gender				0.417			0.469
Male	102	67	35		27	75	
Female	20	15	5		6	14	
Age at diagnosis, years				0.887			0.207
Median (IQR)	63 (39-77)	63 (41-76)	62 (39-77)		64 (40-76)	62 (39-77)	
Smoking status				0.430			0.571
Former smoker	89	58	31		24	65	
Never smoker	33	24	9		9	24	
BMI < 24 kg/m^2^	55	40	15		11	44	0.083
BMI≥24 kg/m^2^	67	42	25		22	45	
Stage				0.032			0.132
LD	71	53	18		16	55	
ED	51	28	22		17	34	
Treatment							
Thoracic radiotherapy				0.001			0.397
No	66	53	13		19	47	
Yes	56	29	27		14	42	
Chemotherapy regimens				0.254			0.571
EP	104	72	32		28	76	
IP	18	10	8		5	13	
Pretreatment laboratory findings							
Median ApoA					1.05 (0.68-1.53)	1.26 (0.66-2.44)	< 0.001
Median ApoB	0.97 (0.26-4.88)	0.98 (0.59-4.88)	0.97 (0.26-2.03)	0.181	1.16 (0.86-4.88)	0.88 (0.26-1.31)	< 0.001
Median ApoB/ApoA-I	0.79 (0.32-4.03)	0.73 (0.32-4.03)	0.96 (0.39-1.93)	< 0.001			
Median lipoprotein (a)	259.4 (6.06-1, 619.8)	261.8 (6.06-1, 619.8)	236 (45-1, 511)	0.987	352.43 (29-1, 511)	227.9 (6.06-1, 619.8)	0.057
IQR: interquartile range; LD: limited-stage disease; ED: extensive-stage disease; EP: etoposide and cisplatin; IP: irinotecan and cisplatin; Apo: apolipoprotein; BMI: body mass index.							

### 2.2 各项指标最佳临界值

用X-tile确定各项指标的最佳临界值，ApoA-I的最佳临界值为1.12 g/L。根据该临界值，将患者分为高组或低组。高位组的ApoA-I > 1.12 g/L，低位组的ApoA-I≤1.12 g/L。ApoB的临界值为0.96 g/L和1.26 g/L。参考临界值，将患者分为高组（ApoB > 1.26 g/L），中组（1.26 g/L≥ApoB > 0.96 g/L）和低组（ApoB≤0.96 g/L）。参考ApoB/ApoA-I的临界值（0.97），将患者分为高组（ApoB/ApoA-I > 0.97）或低组（ApoB/ApoA-I≤0.97）。脂蛋白a的最佳临界值为288 mmol/L。患者分为高组（脂蛋白a > 288 mmol/L）或低组（脂蛋白a≤288 mmol/L）。

### 2.3 不同ApoA-I分组患者的临床特征

对不同ApoA-I分组的患者的临床特征进行比较，治疗前的血清ApoA-I水平与分期（*P*=0.032）和胸部放疗史（*P*=0.001）相关，并且两组间患者ApoB/ApoA-I水平的差异有统计学意义（*P* < 0.001）（表 1）。两组之间的年龄、性别、吸烟状况和治疗方案的差异均无统计学意义（*P* > 0.05）。

### 2.4 不同ApoB/ApoA-I分组患者的临床特征

对于不同ApoB/ApoA-I分组患者之间的临床特征进行比较，结果发现不同ApoB/ApoA-I分组之间的ApoA、ApoB水平有明显差异（*P* < 0.001），并且ApoB/ApoA-I水平与BMI具有相关性，尽管这种差异并不明显（*P*=0.083）。两组患者其他临床特征之间的差异均无统计学意义（表 1）。

### 2.5 生存分析和单因素*Cox*回归分析

各项指标以PFS和OS两个不同研究终点分别进行单因素分析，结果发现具有较高ApoA-I的患者的中位PFS时间比具有较低ApoA-I的患者更长（7.3个月*vs* 5.5个月，*P*=0.017），用*Kaplan-Meier*法绘制生存曲线（图 1A）。单因素*Cox*回归分析显示两组患者之间的PFS有统计学差异（*P*=0.018, HR=0.63, 95%CI: 0.43-0.93）。ApoA-I高组的患者（ApoA-I > 1.12 g/L）的中位OS时间比ApoA-I低组患者要长（21.5个月*vs* 12.3个月，*P*=0.007）（图 1B），单因素*Cox*回归分析显示*P*=0.008（HR=0.52, 95%CI: 0.32-0.84），两组患者的OS差异具有统计学意义。相反，ApoB/ApoA-I高组患者的中位OS短于ApoB/ApoA-I低组（13.4个月*vs* 20.7个月，*P*=0.012）（图 1C）。单变量*Cox*回归分析显示两组OS之间差异有统计学意义（*P*=0.013, HR=1.85, 95%CI: 1.14-3.03）（表 2）。

**1:**
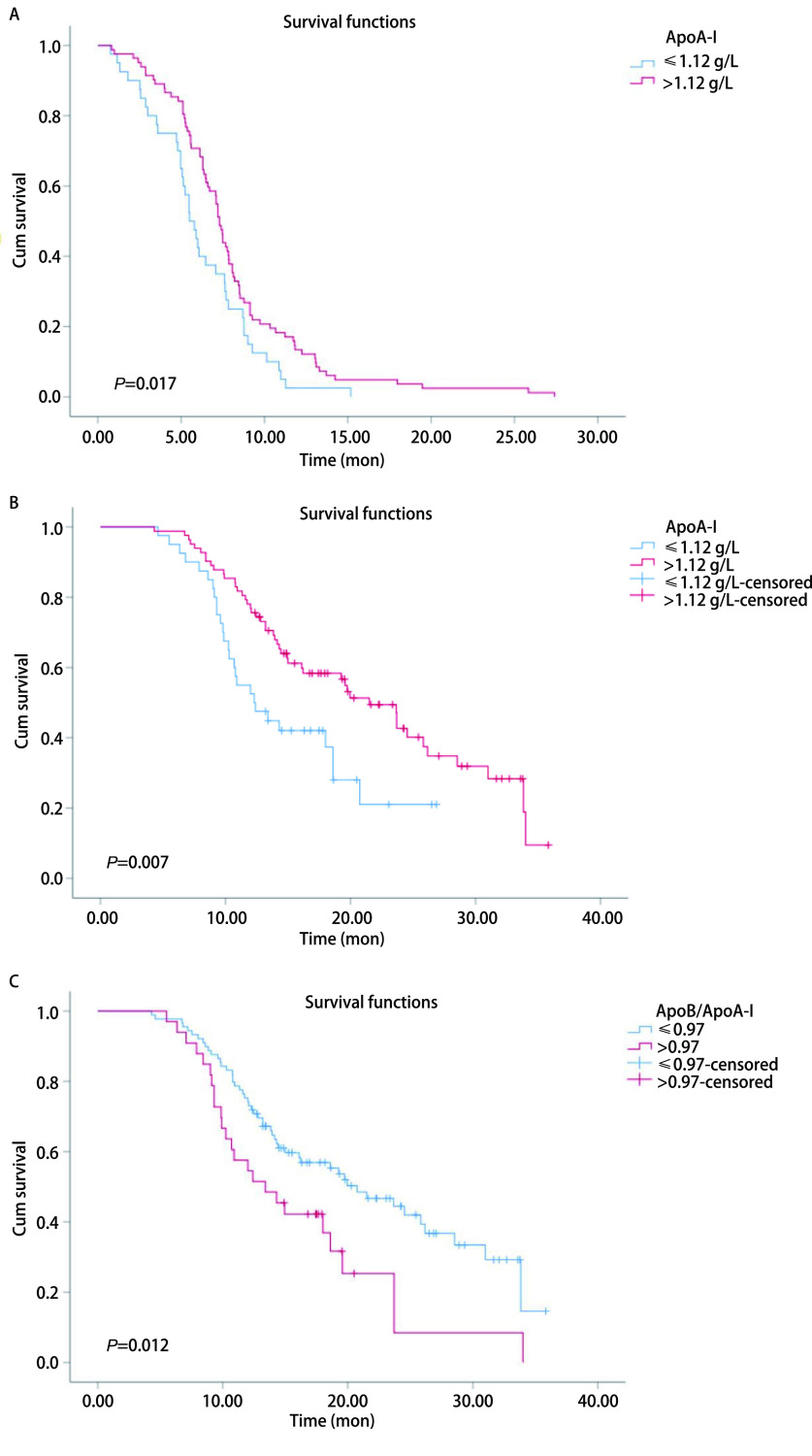
生存曲线。A：无进展生存期：不同ApoA-I分组患者的生存曲线；B：总生存期：不同ApoA-I分组患者的生存曲线；总生存期：不同ApoB/ApoA-I分组患者的生存曲线。 *Kaplan-Meier* curves. A: Progression free survival: *Kaplan-Meier* curves of patients with different ApoA-I groups; B: Overall survival: *Kaplan-Meier* curves of patients with different ApoA-I groups; C: Overall survival: *Kaplan-Meier* curves of patients with different ApoB/ApoA-I groups.

**2:** 可能相关因素的单因素分析和多因素分析 Univariate analysis and multivariate analysis of factors potentially associated

Characteristics	Univariate analysis of factors potentially associated								Multivariate analysis of factors potentially associated						
	PFS				OS				PFS				OS		
	HR	95%CI	*P*		HR	95%CI	*P*		HR	95%CI	*P*		HR	95%CI	*P*
Age≥61 yrs	0.85	0.57-1.26	0.422		0.91	0.55-1.5	0.704								
Female	0.84	0.52-1.36	0.469		1.08	0.61-1.9	0.792								
BMI	0.87	0.61-1.26	0.464		1.45	0.92-2，29	0.107								
Former smoker	1.68	1.12-2.53	0.012		1.84	1.04-3.26	0.035								
Stage	1.71	1.18-2.48	0.004		1.96	1.24-3.09	0.004								
Thoracic radiotherapy	0.41	0.28-0.59	< 0.001		0.45	0.28-0.72	0.001		0.42	0.45-0.99	< 0.000, 1		0.43	0.27-0.7	0.001
Chemotherapy regimens	1.46	0.88-2.42	0.14		1.77	1-3.13	0.049								
ApoA-I	0.63	0.43-0.93	0.018		0.52	0.32-0.84	0.008		0.67	0.45-0.99	0.043		1.98	1.21-3.23	0.007
ApoB	1.08	0.83-1.43	0.583		1.23	0.88-1.72	0.223								
ApoB/ApoA-I	1.43	0.95-2.14	0.085		1.85	1.14-3.03	0.013								
Lipoprotein (a)	1.02	0.71-1.47	0.898		1.03	0.65-1.63	0.918								
PFS: progression-free survival; OS: overall survival.															

### 2.6 多因素*Cox*回归分析

单因素生存分析显示影响患者预后的变量（吸烟史、分期、胸部放疗史、ApoA-I和ApoB/ApoA-I）纳入多因素*Cox*回归模型以进行风险分析。结果显示胸部放疗史（HR=0.42, 95%CI: 0.45-0.99, *P* < 0.000, 1）和高水平ApoA-I（HR=0.67, 95%CI: 0.45-0.99, *P*=0.043）为SCLC患者PFS的独立预后因素（表 2）。胸部放疗史（HR=0.43, 95%CI: 0.27-0.70, *P*=0.001）和ApoB/ApoA水平（HR=1.98, 95%CI: 1.21-3.23, *P*=0.007）可作为SCLC患者OS的独立预后因素（表 2）。

## 3 讨论

脂质在恶性肿瘤的发生和发展中起着重要作用，越来越多的证据^[21, 22]^表明载脂蛋白可能与某些癌症的发生或预后有关，例如卵巢癌、膀胱癌、肾癌、结肠癌和胰腺癌。在这项回顾性队列研究中，我们发现治疗前血清ApoA-I水平与SCLC患者的PFS呈正相关，ApoA-I是影响患者PFS的独立预后因素。SCLC患者的OS、ApoB/ApoA-I是独立危险因素，具有较高血清ApoB/ApoA-I水平的患者具有较差的OS。

ApoA-I是HDL的主要结构成分，主要在肝脏和小肠中合成，负责将胆固醇从周围组织转移到肝脏^[23]^。有研究^[24-26]^表明高水平的ApoA-I可显著增加乳腺癌的患病风险，但是反过来可降低肺癌的发病风险。全身炎症反应已被证实广泛参与肿瘤的发生及发展，而ApoA-I作为HDL不可缺少的成分，可抑制单核细胞的驱化和募集，从而参与炎症反应，在成熟的免疫系统中ApoA-I可被激活参与抗肿瘤活动^[27, 28]^。ApoA-I已被证实在体内外的抗肿瘤作用，ApoA-I可有效抑制体内肿瘤的生长及转移，并提高小鼠肿瘤模型的存活率^[29]^。实验室小鼠模型显示ApoA-I具有可通过多种免疫调节机制来发挥抗肿瘤作用，主要包括：促肿瘤的M2巨噬细胞向抗肿瘤的M1表型转化，肿瘤被细胞毒性的CD8^+^ T细胞浸润，抗血管生成，MMP-9的活性以及survivin的表达^[30]^。ApoB是一种将脂质（包括胆固醇和甘油三脂）运输到肝外组织的载体，有报道^[24, 25, 31]^称高水平的ApoB可增加结肠癌及肺癌的发病风险而降低女性乳腺癌的发病风险。我们研究的结果显示具有较高ApoB/ApoA-I水平的患者有较差的OS，这与其他关于ApoB/ApoA-I与癌症的关系的研究具有一致性，一项美国多种族人群的前瞻性研究^[32]^显示ApoB/ApoA-I水平和ApoB水平显著预测了癌症死亡率，并且独立于几个心脏代谢的危险因素。具有较高ApoB/ApoA-I水平和较高ApoB水平的个体可能具有较高的癌症死亡风险。我们推测，ApoB/ApoA-I对预后的影响可能与ApoA-I的保护作用以及ApoB对癌症的有害影响有关。但是，我们的结果表明，SCLC患者的血清ApoB水平与PFS和OS之间没有显著的相关性，这表明高水平的ApoB可能是SCLC患者的危险因素，由于我们研究的样本量小且随访时间短，可能无法证实这一推测，ApoA-I和ApoB影响SCLC患者的具体机制尚需进一步研究和证实。

这项研究也有一定的局限性，我们纳入的所有患者均来自单一机构，样本量较小，需要更多的前瞻性随机多中心研究来进一步证实我们的结论。其次，应补充其他血清胆固醇指标以提高结论的可靠性。
